# Surgical management of a large cystic trochlear nerve schwannoma mimicking a brainstem glioma: a case report

**DOI:** 10.3389/fonc.2024.1474372

**Published:** 2024-11-11

**Authors:** Miroslav Fimic, Patrick Haas, Jose Antonio Ortiz Rey, Marcos Tatagiba

**Affiliations:** ^1^ Department of Neurosurgery, Tübingen University Hospital, Tübingen, Germany; ^2^ Department of Pathology, Hospital Álvaro Cunqueiro, Vigo, Spain

**Keywords:** trochlear nerve, schwannoma, neurinoma, surgery, case report

## Abstract

**Introduction:**

Intracranial schwannomas represent a rare group of intracranial tumors, with purely motor nerve schwannomas being the rarest of them. The anatomical proximity of these tumors to the brainstem may present a radiological challenge in differentiating them from intra–axial brainstem tumors, which can influence further decision–making and treatment options.

**Methods:**

We report on a 47–year–old male patient who was diagnosed with a large cystic intracranial tumor with radiological features suggestive of an intrinsic brainstem glioma.

**Results:**

After discussing treatment options and risks based on a presumed radiological diagnosis, microsurgical treatment via lateral–suboccipital craniotomy in semi–sitting position, under continuous intraoperative neuromonitoring was performed. Intraoperative findings proved that the tumor was an extra–axial schwannoma originating from the left trochlear nerve. Gross total removal of the lesion was achieved.

**Conclusion:**

Due to their rarity, non–specific symptoms and the possibility to mimicking intra–axial brainstem tumors on imaging, these tumors may present a diagnostic challenge and should be taken into account during treatment decision-making.

## Introduction

Intracranial schwannomas represent approximately 8% of central nervous system (CNS) tumors, representing a relatively uncommon group (1). Non-vestibular schwannomas of purely motor nerves, such as the trochlear nerve, are considered to be the rarest of all cranial nerve schwannomas (1, 27). Due to their proximity to the brainstem, radiological differentiation between trochlear nerve schwannomas and intrinsic brainstem gliomas can sometimes be challenging, potentially influencing decision-making concerning treatment. We present a case of a rare cystic trochlear schwannoma with imaging features suggestive of a brainstem glioma.

## Case presentation

A 47-year-old male patient, who himself works as a medical colleague in pathology, presented at our center in November 2012. He had been complaining of dizziness when turning his head for eight months, which lasted for a few seconds at a time. Climbing stairs with turns or lying down quickly in bed were trigger factors. Additionally, he reported paresthesia of the left cheek corresponding to the maxillary branch of the trigeminal nerve and some minor difficulties in swallowing. Patient’s past medical history was unremarkable except for mild asthma. Family medical history was positive for lattice (reticular) corneal dystrophy that required corneal transplantation and lung cancer from mother’s side, as well as arterial hypertension and diabetes type II from father’s side.

**Figure 1:**
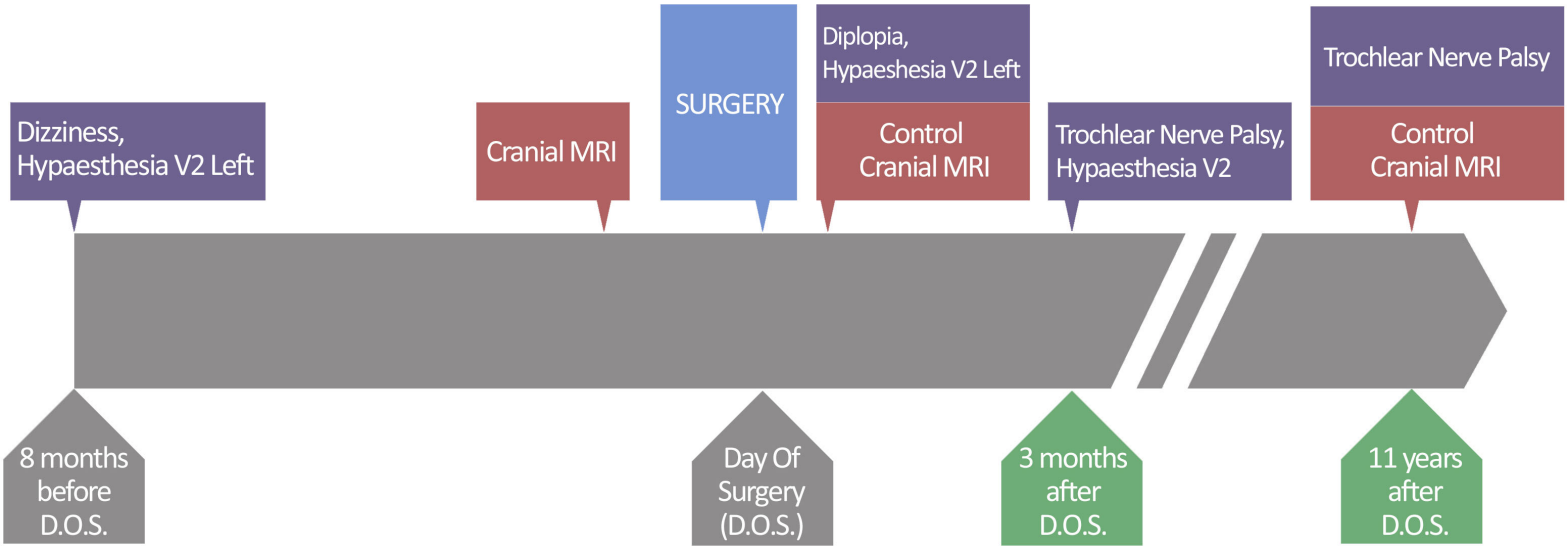
Timeline showing clinical course (purple boxes) since the beginning of the symptoms 8 months before surgery, diagnostic work-up (red boxes), treatment (blue box) and follow-up (green boxes).

A neurologic examination revealed hypoesthesia in the area of the left V2 and unsystematic dizziness. The status of other cranial nerves was intact. No motor or sensory long-tract signs were observed. Muscle reflexes were symmetric, Babinski sign was negative and the finger–to nose–test was without evidence of dysmetria. The patient´s gait was unimpaired (**Figure 1**).

**Figure 2:**
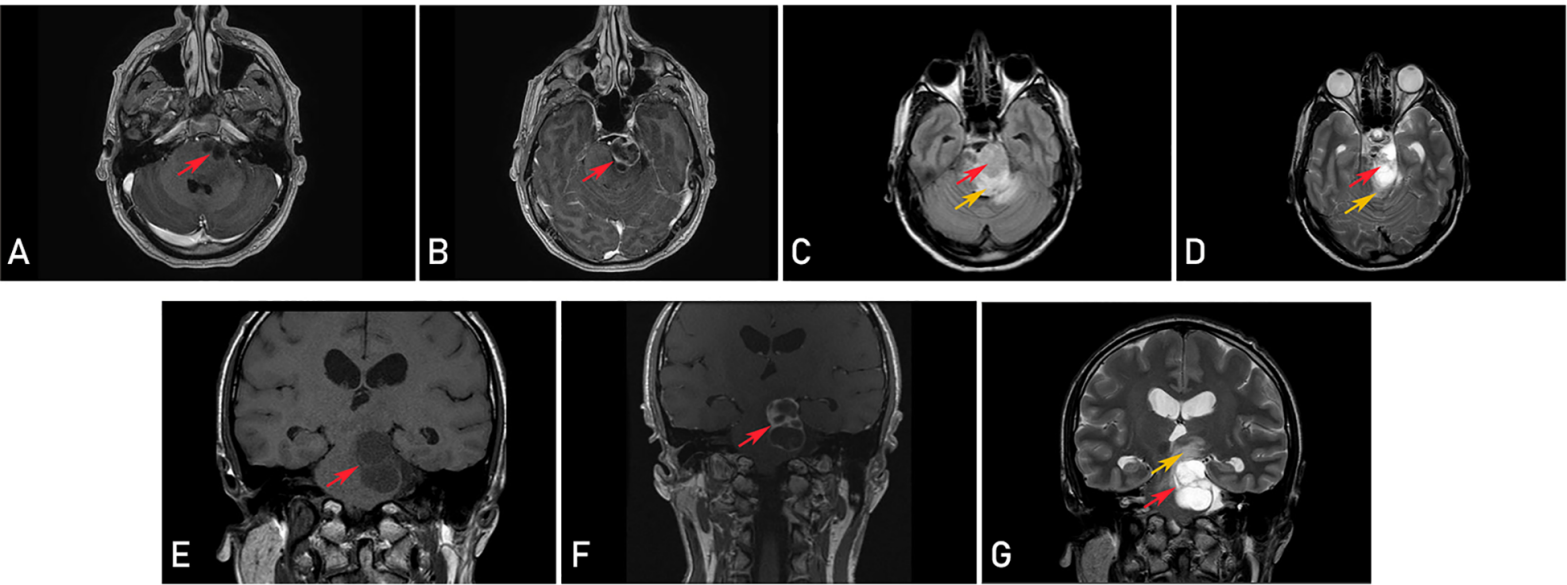
Preoperative magnetic resonance imaging (MRI) revealing a cystic, inhomogeneously enhancing tumor (red arrows) and perifocal vasogenic edema (orange arrows) in axial (T1-weighted contrast-enhanced **(A, B)**, T2-fluid attenuated (FLAIR) **(C)** and T2-weighted **(D)** sequences) and coronal plane (T1-weighted **(E)**, T1-weighted contrast-enhanced **(F)** and T2-weighted **(G)** sequences).

A head MRI revealed a cystic, inhomogeneously enhancing tumor that appeared to originate within the brainstem with supra- and infratentorial extension and strong perifocal vasogenic edema. The diagnosis of pilocytic astrocytoma was presumed, and a differential diagnosis of cystic cranial nerve schwannoma was considered (**Figure 2**). Treatment options and risks based on the suspected diagnosis were discussed with the patient. The decision was made to proceed with a microsurgical removal of the lesion under continuous intraoperative neuromonitoring.

The operation was conducted in a semi-sitting position. Following the retrosigmoid craniotomy, the dura mater was incised under the microscope, along the transverse and sigmoid sinus. The cerebellomedullary cistern was opened and cerebrospinal fluid (CSF) was released, which lowered the pressure in the posterior cranial fossa. After microsurgical preparation of the cerebellopontine angle cistern, the nerve complex VII and VIII were identified, as well as a large cystic structure above them. Xanthochrome fluid was drained from a cyst, and the trigeminal nerve was visualized. It was tightly attached to the yellowish hard tumor capsule. Microsurgical dissection of the tumor capsule from the trigeminal nerve and petrosal vein was then performed. Then the solid part of the tumor was debulked with an ultrasonic aspirator while several tumor pieces were sent for histopathological frozen section examination. The frozen specimen results indicated that the tumor was most likely a schwannoma, thereby excluding the diagnosis of glioma. Following further reduction of the tumor, the trochlear nerve was identified. Neurolysis of the nerve was initiated, but it was observed that the nerve completely dissolved into the tumor. Using a meticulous microsurgical technique, the tumor capsule and the solid part of the tumor were dissected from the oculomotor nerve and the brainstem surface. This procedure resulted in a gross total resection of the tumor with the preservation of all neurovascular structures, except for the trochlear nerve, which was thought to be the source of the tumor. Based on these findings, the intraoperative diagnosis of the cystic trochlear nerve schwannoma was established.

Postoperatively, the patient reported diplopia, which improved during the course of the hospital stay. A surveillance MRI confirmed the gross total resection of the tumor and signs of a resolving edema in the brainstem. The final histopathologic examination revealed tumor tissue interspersed with nerve fibers exhibiting the typical features of a WHO grade I schwannoma (**Supplementary Image**).

Three months following surgery the patient reported the persistence of hypoesthesia in the V2 segment of the trigeminal nerve, though with a decreasing tendency. The examination confirmed the persistence of trochlear nerve palsy, which was satisfactorily compensated by prismatic lenses.

**Figure 3:**
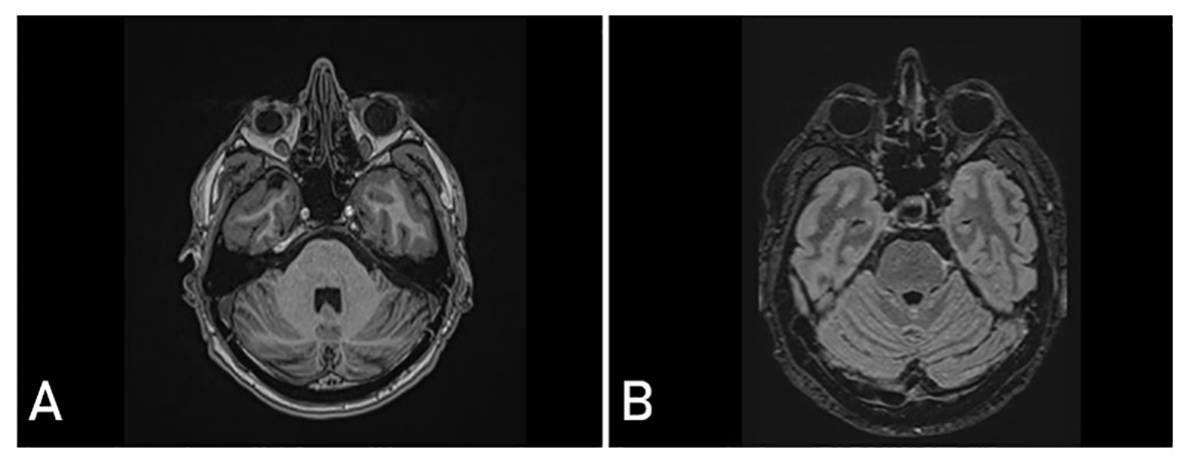
Postoperative magnetic resonance imaging (MRI) 11 years after surgery showing complete realignment of the brainstem and no signs of tumor recurrence in T1-weighted contrast - enhanced **(A)** and T2 fluid attenuated (FLAIR) **(B)** sequences.

A follow-up examination conducted 11 years after the removal of the tumor revealed the persistence of a palsy of the left trochlear nerve. However, the use of prismatic lenses effectively mitigated the issue of double vision. The patient has been able to resume his occupational and daily activities without difficulty. There was no evidence of tumor recurrence on the surveillance cranial MRI (**Figure 3**).

## Discussion

Intracranial schwannomas are benign tumors that arise from Schwann cells in the nerve sheath of cranial nerves and represent approximately 8% of all intracranial tumors (1). Most commonly they arise from sensory nerves or mixed motor–sensory nerves (1). In the majority of the cases, there is an association between intracranial schwannomas and neurofibromatosis type 2 (2). Purely motor nerve schwannomas, especially sporadic, are rare, and schwannomas originating from the trochlear nerve are considered to be the rarest (3).

A literature search of the PubMed and Web of Science databases was conducted using the following terms: “trochlear nerve”, “tumor”, “schwannoma”, “neurinoma” and “surgery”. The search was limited to English-language sources published between 1976 and 2024 (**Supplementary Table 1**). This yielded a total of 45 documented cases of surgically treated trochlear nerve schwannomas with histological confirmation. The median age was 44 years, with age at time of treatment ranging from 12 to 72 years. The majority of patients were in the fifth (23.9%), sixth (21.7%) or seventh (21.7%) decade of life. There was no difference in gender distribution (male-to-female ratio 1.09:1).

Celli et al. categorized trochlear nerve schwannomas into three groups: cisternal, cisternocavernous and cavernous (2). The majority of trochlear schwannomas are of the cysternal type and are located in the ambient cistern. However, in four cases, the tumor was located in the pineal region (3, 4, 8, 10, 11). Large cisternal type trochlear schwannomas typically extend supra- and infratentorial, exerting compression on the brainstem and adjacent cranial nerves, which contributes to the variety of unspecific symptoms they cause (12). In the literature, the most common symptoms are diplopia, headache and ataxia. Other reported symptoms included focal neurological deficits, including long tract signs or other cranial nerves palsies (mostly cranial nerves V and VII), tinnitus, vertigo and hearing loss (8). Cases of unusual presentation such as pathological laughter, atypical facial pain or persistent hiccups were also described (13–15). In cases complicated by intratumoral hemorrhage, signs of increased intracranial pressure, such as nausea and vomiting, were observed. Additionally, sudden onset or acute exacerbation of preexisting symptoms was noted (9, 12, 15–17).

Intracranial schwannomas exhibit distinctive characteristics on MRI. On T2–weighted images, they appear as a heterogeneously hyperintense lesions. They appear as a low or intermediate signal lesions on T1–weighted images, with avid enhancement after intravenous contrast administration (18) (**Supplementary Table 2**).

Although not so common like in skull base meningioma, presence of peritumoral brain edema (PTBE) in patients with vestibular schwanommas (VS) is also reported in the literature (5 – 10%). Since the mechanism of formation is still unclear, it is the topic of ongoing research. Direct compression of the brainstem leading to a decrease of cerebral blood flow and a slowed metabolism of nearby cells can cause cytotoxic edema. On the other hand, some research stated that PTBE was actually vasogenic brain edema. Hong-Hai You et al. reported about strong relationship among Vascular Endothelial Growth Factor (VEGF) expression, tumor angiogenesis, and PTBE formation in patients with VS (29).

In our review, the presence of a cyst was reported in 22 cases. Due to their proximity to the brainstem, cisternal schwannomas may appear on imaging as intrinsic brainstem lesion (5, 6). This may lead to the diagnostic uncertainty and influence decision-making concerning the appropriate treatment. In our case, according to the preoperative radiographic findings, intrinsic brainstem glioma was suspected. The presumed differential diagnosis was cystic cranial nerve schwannoma. Considering the eloquent location of brainstem gliomas, the therapy of choice in many cases is biopsy for histological confirmation followed by radiation in case of progression (24). In our case the decision was made to remove the tumor, which proved to be a correct decision given that the tumor was extra-axial and benign and a gross total resection was achieved. Conversely, cases of intrinsic brainstem glioma with imaging characteristics of a cranial nerve schwannoma have also been reported (7, 24).

In the literature, the most commonly utilized approach for the resection of the trochlear nerve schwannoma is the subtemporal transtentorial (32.6%) approach, followed by a lateral - suboccipital approach (26.1%) (8). In addition, pterional and anterior or posterior transpetrosal approaches were described, as well as transventricular transvelar and paraoccipital posterior interhemispheric transtentorial approaches to the lesions in the pineal region (3, 11, 15, 19–21). To the best of our knowledge, this is the first reported case of a cystic trochlear nerve schwannoma that has been operated via the lateral-suboccipital approach in the semi-sitting position, an approach that has been demonstrated to be an effective technique for the removal of large tumors, resulting in improved tumor resection and nerve preservation (22).

The trochlear nerve is the sole cranial nerve to emerge from the brainstem on the dorsal side and crosses the midline. Additionally, it is the longest and the thinnest of all cranial nerves (12). Despite its inherent vulnerability to injury, trochlear nerve palsy was observed preoperatively in only 21 patients (45.6%) in our review. In the majority of cases (82.6%) gross total resection of the tumor was achieved. Trochlear nerve palsy was reported in 33 patients (71.7%) in the postoperative course. Treatment modalities for diplopia include prism lenses and strabismus surgery (25). Fujiwara et al. reported that even some patients whose trochlear nerve was cut intraoperatively did not develop diplopia after surgery. This may be the result of the fusion of the visual fields occurring prior to surgery during tumor progression (23).

Regarding the management of trochlear nerve schwannomas, Torun et al. in their 2018 literature review and the group of authors in their 2022 consensus statement on behalf of the European Association of Neurosurgical Societies (EANS) skull base section propose a multidisciplinary and tailored approach (25, 28). Observation with clinical and radiological follow-up may be sufficient for smaller tumors in patients without major symptoms. Lock et al. reported on a case of a trochlear schwannoma that was not surgically treated. During a 22–year follow-up period, no tumor progression was observed (26). In patients with larger tumors and symptoms caused by mass effect, surgery is the therapy of choice (28). Ozoner et al. reported in their literature review that there was no difference in outcome with respect to subtotal or total tumor resection (8). In our 11–year follow-up, cranial MRI showed no evidence of tumor recurrence with complete realignment of the brainstem and complete resolution of the brainstem vasogenic edema. Farrokhi et al. observed no tumor recurrence in their 12–year postoperative follow-up (3). For small tumors with a tendency to grow or residual tumor, stereotactic radiosurgery may be the therapy of choice, although the potential vulnerability of the trochlear nerve should be considered (27, 28).

## Conclusion

Due to their rarity and non-specific symptoms, trochlear nerve schwannomas may present a diagnostic challenge and should be considered in the differential diagnosis of the unclear expansive lesions of the brainstem and cerebellopontine angle. For large tumors requiring surgical treatment, a lateral-suboccipital approach in a semi-sitting position may offer the possibility of a safe gross total tumor resection and a good outcome.

## Data Availability

The original contributions presented in the study are included in the article/ (**Supplementary Material**). Further inquiries can be directed to the corresponding author.
