# Supplementary figures and images for: Surgical management of a large cystic trochlear nerve schwannoma mimicking a brainstem glioma: a case report
Source: Front Oncol. 2024 Nov 11;14:1474372. doi: 10.3389/fonc.2024.1474372 (PMC11586389; doi:10.3389/fonc.2024.1474372)

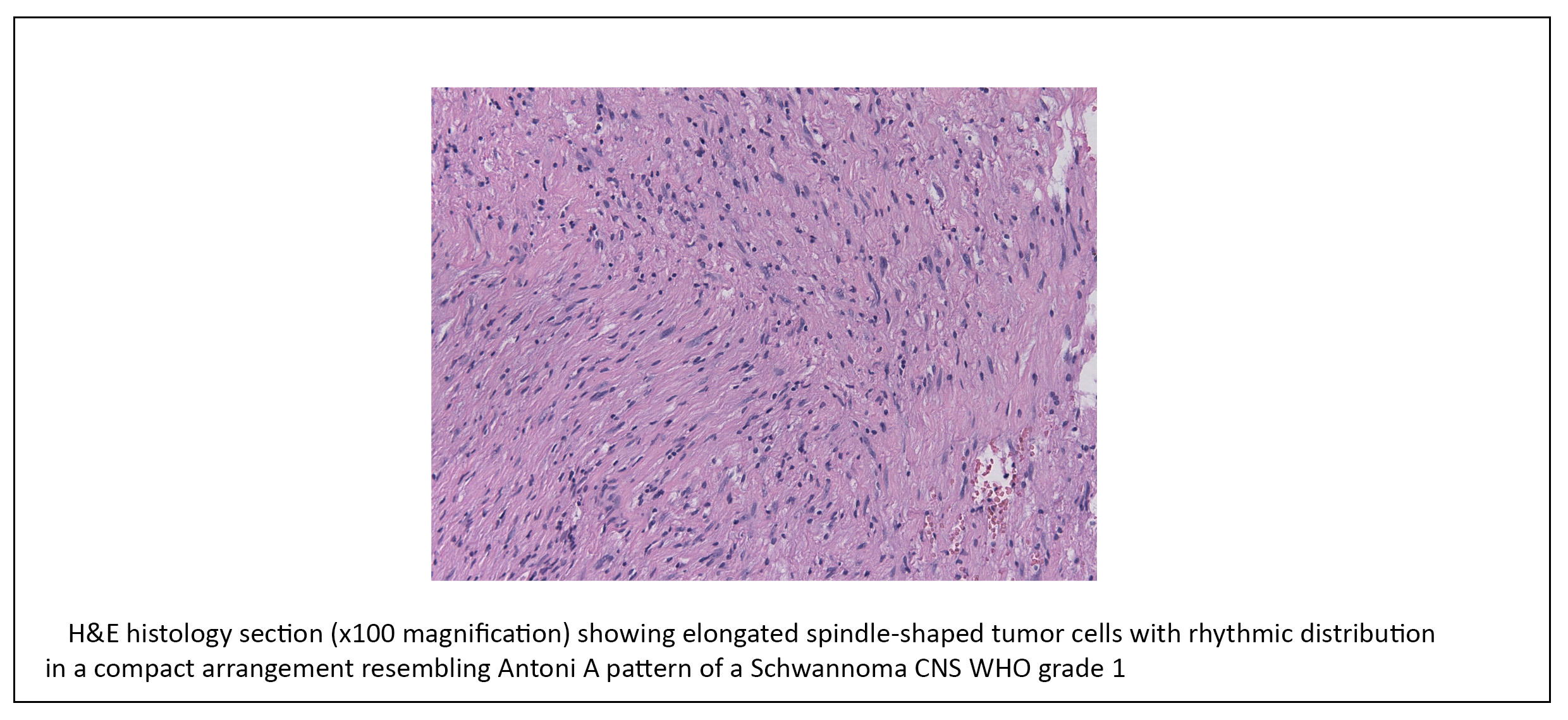

Supplement: Supplementary file 1 [file Image1.jpg]
