# Supplementary material for: Surgical management of a large cystic trochlear nerve schwannoma mimicking a brainstem glioma: a case report
Source: Front Oncol. 2024 Nov 11;14:1474372. doi: 10.3389/fonc.2024.1474372 (PMC11586389; doi:10.3389/fonc.2024.1474372)
Supplement: Supplementary file 3 [file Table2.docx]

**Table 2.** Magnetic resonance imaging (MRI) characteristics of cranial nerve schwannoma vs pilocytic astrocytoma
